# Probenecid Inhibits Influenza A(H5N1) and A(H7N9) Viruses In Vitro and in Mice

**DOI:** 10.3390/v16010152

**Published:** 2024-01-19

**Authors:** Jackelyn Murray, David E. Martin, Sarah Hosking, Nichole Orr-Burks, Robert J. Hogan, Ralph A. Tripp

**Affiliations:** 1 Animal Health Research Center, Department of Infectious Diseases, College of Veterinary Medicine Athens, University of Georgia, Athens, GA 30605, USA; jcrab@uga.edu (J.M.); sarah.hosking@uga.edu (S.H.); snook@uga.edu (N.O.-B.); jhogan@uga.edu (R.J.H.); 2 TrippBio, Inc., Jacksonville, FL 32256, USA; davidmartin@trippbio.com

**Keywords:** highly pathogenic, HPAI, antiviral, avian influenza, H5N1, H7N9

## Abstract

Avian influenza (AI) viruses cause infection in birds and humans. Several H5N1 and H7N9 variants are highly pathogenic avian influenza (HPAI) viruses. H5N1 is a highly infectious bird virus infecting primarily poultry, but unlike other AIs, H5N1 also infects mammals and transmits to humans with a case fatality rate above 40%. Similarly, H7N9 can infect humans, with a case fatality rate of over 40%. Since 1996, there have been several HPAI outbreaks affecting humans, emphasizing the need for safe and effective antivirals. We show that probenecid potently inhibits H5N1 and H7N9 replication in prophylactically or therapeutically treated A549 cells and normal human broncho-epithelial (NHBE) cells, and H5N1 replication in VeroE6 cells and mice.

## 1. Introduction

Despite HPAI H5 and H7 vaccines, which can lower the burden of human infection, having an effective antiviral to bridge efficacy is important. Therapeutics against these HPAI viruses are among the highest priority areas for influenza research. Probenecid, a uricosuric agent approved in 1951 to treat gout, was later found to have potent, broad-spectrum antiviral activity against several respiratory viruses. Accumulating studies from our lab have shown that probenecid has potent antiviral effects, specifically inhibiting the replication of SARS-CoV-2 variants, RSV, and contemporary influenza strains both in vitro and in vivo [1,2,3,4,5,6]. This study shows that probenecid has robust antiviral effects, inhibiting HPAI H5N1 and H7N9 replication, and can reduce serum pro-inflammatory cytokine expressed in response to infection.

H5N1 low pathogenic avian influenza (LPAI) strains occur in wild birds, causing minor sickness or no noticeable signs of disease, and are not known to affect humans. In contrast, HPAI H5N1 may cause severe human disease with a high mortality rate [7]. The HPAI H5N1 virus must contain a multi-basic amino acid R-X-R/K-R motif in the HA protein, which can be proteolytically activated by ubiquitous subtilisin-like cellular proteases [8]. HPAI H5N1 can spread systemically beyond the respiratory tract and cause multi-organ failure [9]. The first human outbreak of H5N1 occurred in Hong Kong in 1997; subsequently, there have been outbreaks in different parts of the world. For example, since 2003, there have been over 900 cases of human infection with H5N1 across 23 countries, of which 450 were fatal [10]. Another important AI strain is H7N9. In March 2013, three patients were hospitalized with severe lower respiratory tract infections of unknown origin [11]. By May 2013, China had reported 132 confirmed H7N9 infections and nearly 40% of those infected with H7N9 died [12]. This novel H7N9 strain was the first LPAI virus documented to have caused severe human disease after it evolved into an HPAI virus. This H7N9 reassortant AI was isolated from respiratory specimens obtained from patients [11]. The genetic characteristics were of concern because of their pandemic potential, e.g., their potential to recognize human and avian influenza virus receptors, which affects their ability to cause sustained human-to-human transmission and replication in humans. HPAI H7N9 had the increased ability to bind to sialic acid receptors [13] and had several mutations in the PB2 protein (e.g., T271A, K526R, E627K, or D701N) that were associated with increased virus replication rates and disease severity in mammals and/or humans [14]. For these reasons, H7N9 is considered by the WHO to be a serious human virus of concern. Together, H5N1 and H7N9 have caused hundreds of severe or fatal human cases [7,15]. Fortunately, the U.S. government maintains a stockpile of H5N1 and H7N9 vaccines. For example, H5N1 A/Vietnam/1203/2004 (VN1203-H5N1) was manufactured and stockpiled as both a bulk antigen and multi-dose final container vaccine [16], while a recombinant VLP (A/Anhui/1/2013) containing 15 μg hemagglutinin (HA) + ISCOMATRIX was stockpiled [17]. Although vaccines have been developed, the current H5N1 virus has since mutated, which might make the vaccines ineffective [18]. Newer H5N1 and H7N9 vaccine candidates are being investigated to protect against a potential pandemic. For example, two reassortant influenza vaccines expressing H5 or H7 HAs (PR8-H5-H7NA and PR8-H7-H5NA) have been shown to replicate to high titers when exposed to exogenous neuraminidase (NA) in vitro, and these vaccine candidates were shown to be replication-defective and nonvirulent when administered intranasally in mice [19]. Vaccination with PR8-H5-H7NA elicited robust immune responses to H5 and H7 viruses and conferred complete protection against H5N1 and H7N9 challenges in mice. Thus, PR8-H5-H7NA has the potential to serve as a vaccine candidate against both H5 and H7 subtypes of the HPAI viruses. Despite HPAI H5 and H7 vaccines, having an effective antiviral to bridge efficacy is important. HPAI H5N1 viruses may be susceptible to neuraminidase inhibitors (NAI), e.g., oseltamivir, peramivir, zanamivir, and the endonuclease inhibitor (CENI) baloxavir marboxil, but this has not been wholly substantiated. NAI treatments with oseltamivir, peramivir, or zanamivir have been used for severely ill persons infected with H7N9 viruses, but their effectiveness for treating severe disease has not been determined [11,20,21]. Moreover, studies show that H5N1 has acquired resistance to some of these therapeutics [22,23]. Therefore, therapeutics against these HPAI viruses are among the highest priority areas for influenza research, and the search for antiviral drugs continues.

Beyond HPAI H5N1 and H7N9, disease outbreaks caused by respiratory viruses are a major public health concern, as exemplified by the COVID-19 pandemic that has claimed > 6 million lives [24]. Direct-acting antiviral therapeutics target viral components, whereas host-directed antivirals interfere with cellular gene expression and pathways [25,26]. Probenecid is a host-directed antiviral (HDA) drug that inhibits virus replication by inhibiting a necessary host cell pathway [27]. This is relevant in pandemic preparedness, where the goal is to identify active antiviral agents; as such, screening efforts should be sufficiently robust to identify all potential targets regardless of the antiviral mechanism.

Targeting host factors rather than viral components to inhibit viral replication often increases the threshold of viral resistance and can provide broad-spectrum antiviral action against different viruses. An ideal antiviral target is essential for viral replication, has a responsive mechanism of action that can be inhibited, and is averse to drug resistance [28]. In a search for antiviral gene targets, we use genome-wide RNA interference (RNAi) screens to identify host genes and pathways that viruses use for replication [29,30,31,32], and this has led to drug repurposing of probenecid to inhibit SARS-CoV-2, influenza, and RSV replication [3,4,5,6]. Studies from our lab have shown that organic anion transporter-3 (OAT3) is needed to replicate influenza A and B viruses and that probenecid inhibits OAT3 [6]. Probenecid can also inhibit phosphorylation of c-jun N-terminal kinases (JNK) and downstream phosphorylation of the canonical JNK substrate [33], c-jun, a critical component of the activator protein-1 (AP-1) transcription complex needed for virus replication in A549 cells. The inhibition of JNK activity by probenecid is associated with the accumulation of the transcription factor hepatocyte nuclear factor-4 (HNF-4). HNF4 is also regulated at the post-transcriptional level by the extracellular signal-regulated kinase (ERK) [34]. HNF4 has been shown to regulate OAT3 expression [35], and OAT3 is necessary for influenza replication. Thus, probenecid inhibits JNK phosphorylation and JNK, as well as mitogen-activated protein kinases (MAPK) that also inhibit virus replication.

Probenecid belongs to a class of drugs known as uricosurics. Accumulating studies from our lab have shown that probenecid has potent antiviral effects, specifically inhibiting replication of SARS-CoV-2 and variants, as well as influenza and RSV virus strains in vitro and in vivo [4]. Probenecid inhibited the replication of SARS-CoV-2 variants (Beta, Gamma, Delta, and Omicron, B.1.1) in VeroE6 cells and NHBE cells at nanomolar concentrations [5]. Probenecid inhibited RSV and influenza A replication in human respiratory epithelial cell lines [3,6]. Perhaps more importantly, in a Phase 2 probenecid dose-range finding study in non-hospitalized patients with symptomatic, mild-to-moderate COVID-19, the median time to viral clearance was significantly shorter for the 500 mg and 1000 mg probenecid-treated patients (9 vs. 7 days, respectively; *p* < 0.0001) compared to placebo-treated patients (day 11; *p* < 0.0001) [2]. Furthermore, a significantly greater proportion of patients receiving probenecid 1000 mg reported complete resolution of symptoms versus placebo (68% vs. 20%, respectively; *p* = 0.0006), and probenecid 500 mg versus placebo (56% vs. 20%, respectively, *p* = 0.0087). Together, these data showed a significant, dose-dependent decrease in the time to viral clearance and a significantly higher proportion of patients reporting complete symptom resolution by day 10 [2]. Existing antiviral drugs (NAIs and CENI) target conserved amino acids in the NA enzyme and polymerase acidic (PA) protein, respectively [24]. They exhibit antiviral activity against a broad range of influenza A virus subtypes and lineages of influenza B viruses. As probenecid has robust antiviral effects on several respiratory viruses, the effects of drug treatment to inhibit H5N1 and H7N9 replication were investigated due to its pan-antiviral nature.

## 2. Materials and Methods

### 2.1. Ethics

All procedures were approved by the University of Georgia Biosafety Committee and the Animal Care and Use Committee in compliance with the Guide for the Care and Use of Laboratory Animals, 8th edition (Library of Congress Control Number: 2010940400). The animal study protocol was approved by the Institutional Review Board of the University of Georgia, A2021 03-006-Y2-A0, Immunity to Respiratory Viruses and Virus Proteins in Mus musculus, approved on 6 May 2021.

### 2.2. Viruses

A/Vietnam/1203/2004 (VN/1203-H5N1) and A/Anhui/1/2013 (Anhui/1-H7N9) were received from Ted Ross from the University of Georgia, and Richard Webby from St. Jude Children’s Hospital, respectively. VN/1203-H5N1 was originally sourced from the CDC. The viruses were propagated in the allantoic cavity of 10-day-old specific-pathogen-free embryonated chicken eggs at 37 °C and 55–60% humidity. The allantoic fluid was collected, cleared by centrifugation, and stored at −80 °C. Virus titers were determined by 50% tissue culture infective dose (TCID_50_) analysis as previously described [36]. In brief, a virus stock is seeded in triplicate using the Reed and Muench method [37]. The virus is seeded over a MDCK cell monolayer and incubated in the presence of TPCK-trypsin for 72 h at 37 °C and 5% CO_2_. The supernatant is determined by hemagglutination assay [36] to determine end point dilutions. All studies with H5N1 or H7N9 were conducted using BSL3 procedures and facilities at the University of Georgia.

### 2.3. Cells and Virus Infections

A549 cells (ATCC CCL-185, Manassas, VA, USA), VeroE6 cells (ATCC CRL-1586) and Madin–Darby canine kidney (MDCK) cells (ATCC CRL-34) expressing α2,6 sialyl glycans [38] were maintained in Eagle’s minimum essential medium (MEM, Thermofisher, Grand Island, NY, USA) supplemented with 2 mM glutamine and 10% fetal bovine serum (Hyclone, Thermofisher) and grown at 37 °C with 5% CO_2_. The cells were infected at MOI = 0.1 for 1 h at 37 °C and then washed three times to remove the unbound virus, and infected cells were cultured in media containing 1% bovine serum albumin (Sigma, St. Louis, MO, USA) and TPCK (tolyl sulfonyl phenylalanyl chloromethyl ketone; 1 μg/mL)-treated trypsin (Thermofisher).

NHBE cells (Lonza Biosciences, Greenwood, SC, USA) are primary human cells maintained at an air–liquid interface at 37 °C with 5% CO_2_. NHBE cells were seeded at 10,000 cells/cm^2^ on polycarbonate transwell inserts with a 0.4 µm pore size diameter (Costar, Sigma Aldrich, St. Louis, MO, USA) with bronchial epithelial basal medium (BEBM, Lonza) supplemented with 5 μg/mL insulin, 0.5 ng/mL hEGF, 0.5 μg/mL hydrocortisone, 0.5 μg/mL epinephrine, 50 μg/mL gentamycin, 50 μg/mL amphotericin B, 10 μg/mL transferrin, 6.5 ng/mL triiodothyronin, and 0.13 mg/mL bovine pituitary extract (all supplied by Lonza) to obtain bronchial epithelial growth medium (BEGM).

### 2.4. Probenecid and Oseltamivir

Probenecid (CAS Number: 57-66-9) (Sigma, St. Louis, MO, USA) was diluted in DMSO (Sigma, St. Louis, MO, USA) and resuspended in PBS (Gibco, ThermoFisher, Waltham, MA, USA) and oseltamivir carboxylate (Sigma, St. Louis, MO, USA), the active metabolite of oseltamivir phosphate (Tamiflu). They were examined for their inhibitory effect on H5N1 and H7N9 viruses.

### 2.5. In Vitro Assays

A549 cells, NHBE, or Vero E6 cells were plated overnight at 10^4^ cells/well in 96-well flat-bottom plates (Costar, Sigma Aldrich, St. Louis, MO, USA). Cells were pretreated for 24 h before infection or post-virus infection at 1 hpi with probenecid or oseltamivir at different concentrations, i.e., 100,000, 10,000, 1000, 100, 10, 1, 0.1, 0.01, 0.001, or 0 μM. For post-virus-treated cells, the media and probenecid were removed, and the cells were infected with H5N1 or H7N9 at MOI = 0.1.

### 2.6. Mouse Studies

BALB/c female mice (6–8 weeks old) were obtained from Charles River and rested a week before use. The animal study protocol was approved by the Institutional Review Board of the University of Georgia, A2021 03-006-Y2-A0, Immunity to Respiratory Viruses and Virus Proteins in Mus musculus, approved on 6 May 2021. All experiments and procedures were approved by the Institutional Animal Care and Use Committee (IACUC) of the University of Georgia. All experiments were performed with five female mice per group, and the studies were repeated twice independently. Intranasal (i.n.) infections were performed using 3LD_50_ of H5N1. To evaluate lung virus titers, probenecid was administered by gavage at doses and time points pre- or post-virus infection. Briefly, 10 mg/kg of oseltamivir, 10 mg/kg of probenecid, or 100 mg/kg of probenecid in PBS were delivered to the mice twice daily for 3 days pi. The lungs were removed for plaque assay on days 3, 5, and 7 pi [39]. At each time point, sera were collected, and the lungs were isolated to determine virus titers by PFU/mL.

### 2.7. Mouse Serum Cytokine ELISAs

Mouse serum was used to determine the levels of IL-6 (Invitrogen, Thermofisher, KMC0061, Carlsbad, CA, USA), TNF-α (Invitrogen, BMS607-3), IL-1β (Invitrogen, BMS6002), and pannexin-1 (PANX1; Abbexa, Cambridge, UK, abx515359). ELISA kits were pre-coated, and the protocols were followed exactly using the mouse serum. The plates were read at 450 nM wavelength and analyzed. Three independent ELISAs were completed with duplicates in each experiment.

### 2.8. Lung Virus Titers

Lung viral titer was determined as previously described [40]. Briefly, lungs were homogenized in 1 mL of Dulbecco PBS/lung, and 10-fold serial dilutions in serum-free DMEM (Gibco) were added to confluent MDCK cell monolayers in 12-well plates. Following 1 h virus adsorption at 37 °C, 5% CO_2_, 2 mL of overlay containing 1-part medium consisting of 10× MEM supplemented with 200 mM L-glutamine (Gibco), HEPES solution (Gibco), 7.5% NaCHO_3_ (Gibco), Pen/Strep/Amp B solution (Gibco), and 1-part 2.4% Avicel (FMC BioPolymer, Philadelphia, PA, USA) in water was added per well. Samples were incubated at 37 °C, 5% CO_2_ for 3 days and enumerated.

### 2.9. Statistical Analysis

Statistical analyses were performed using the Student’s *t*-test or one-way analysis of variance (ANOVA), as indicated. Data were analyzed for statistical significance using appropriate statistics, where *p* < 0.05 was considered statistically significant using Prism 9 (GraphPad). Results were calculated as means ± standard errors. Values of *p* < 0.05 were considered significant. Nonparametric data were analyzed using a Kruskal–Wallis test (α = 0.05).

## 3. Results

**Figure 1:**
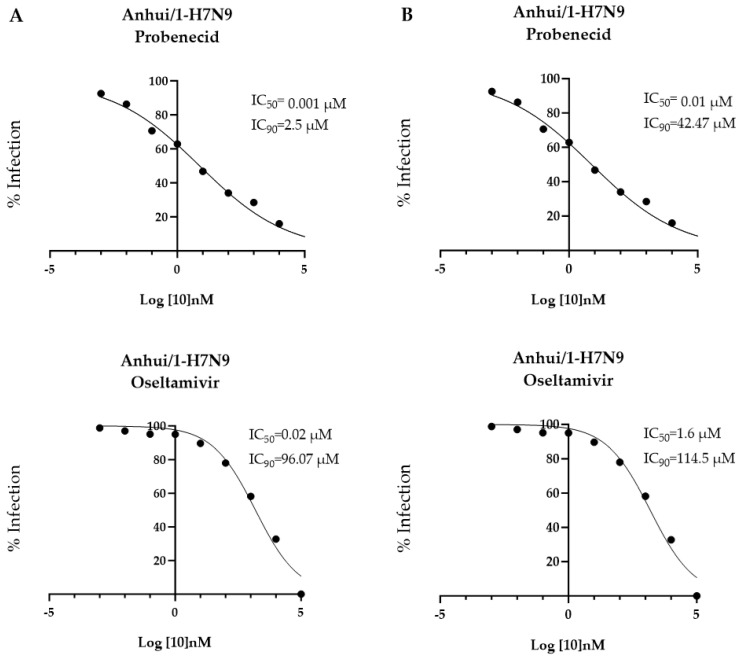
A549 cells were treated 24 h before (**A**) or 1 h after (**B**) inoculation with Anhui/1-H7N9 (MOI = 0.1) with probenecid or oseltamivir at different concentrations (100,000, 10,000, 1000, 100, 1, 0.1, 0.01, 0.001, or 0 µM).

To determine the antiviral efficacy of oseltamivir or probenecid, A549 cells or NHBE cells were treated either (A) 24 h before infection or (B) 1 h after Anhui/1-H7N9 infection. Ten-fold dilutions of probenecid or oseltamivir (100,000, 10,000, 1000, 100, 1, 0.1, 0.01, 0.001, or 0 μM) were examined. The IC_50/90_ for prophylactic treatment with probenecid was 0.001 μM/2.5 μM and 0.02 μM/96.07 μM for oseltamivir treatment. The IC_50/90_ for drug treatment 1 h after infection with probenecid was 0.01 μM/42.47 μM, and 1.6 μM/114.5 μM for the IC_50/90_ for oseltamivir treatment of A/Anhui/2013-infected cells (Figure 1). The IC_50/90_ results show that probenecid inhibited viral replication in A549 cells better than the oseltamivir treatment.

**Figure 2:**
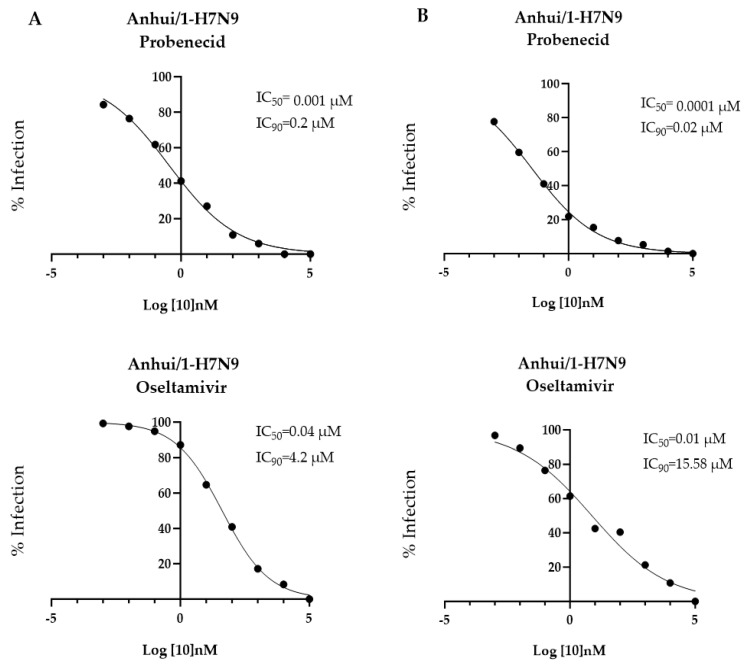
NHBE cells were treated 24 h before (**A**) or 1 h after (**B**) inoculation with Anhui/1-H7N9 (MOI = 0.1) with probenecid or oseltamivir at different concentrations (100,000, 10,000, 1000, 100, 1, 0.1, 0.01, 0.001, or 0 µM).

NHBE cells are a primary human cell line isolated from the epithelial lining of airways above the bifurcation of the lungs. To determine the antiviral effects of probenecid or oseltamivir on NHBE cells, 10-fold dilutions of probenecid or oseltamivir (100,000, 10,000, 1000, 100, 1, 0.1, 0.01, 0.001, or 0 µM) were examined. The IC_50_/IC_90_ for pre-virus infection treatment with probenecid was 0.001 μM/0.2 μM and 0.04 μM/4.2 μM for oseltamivir treatment. The IC_50/90_ for drug treatment 1 h after infection with probenecid was 0.0001 μM/0.02 μM, and 0.01 μM/15.58 μM for the IC_50/90_ for oseltamivir treatment of Anhui/1-H7N9-infected cells (Figure 2). Compared to A549 cells (Figure 1), probenecid inhibited Anhui/1-H7N9 replication, but the efficacy was higher for NHBE cells than oseltamivir treatment of either pre-virus or post-virus infection drug treatment.

**Figure 3:**
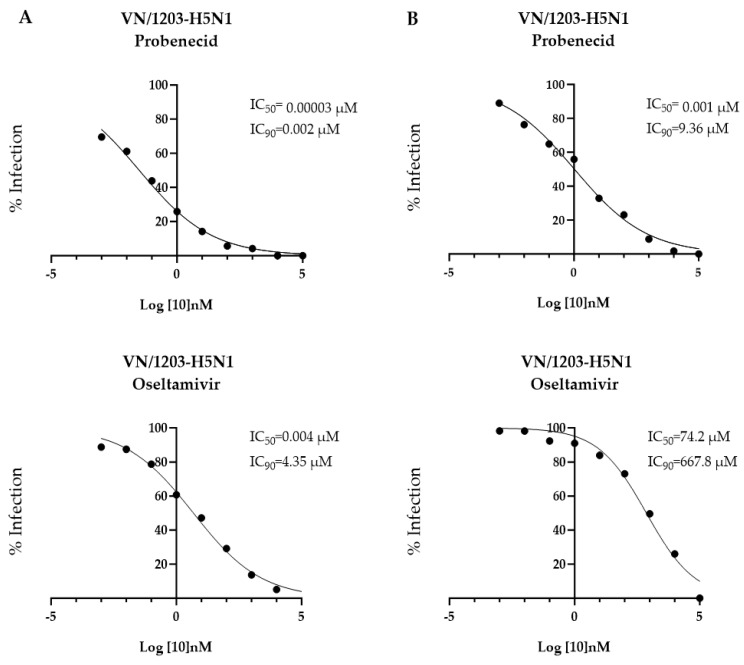
VeroE6 cells were treated 24 h before (**A**) or 1 h after (**B**) inoculation with VN/1203-H5N1 (MOI = 0.1) with probenecid or oseltamivir at different concentrations (100,000, 10,000, 1000, 100, 1, 0.1, 0.01, 0.001, or 0 µM).

Having examined the antiviral effects for Anhui/1-H7N9-infected cells, the antiviral effects of probenecid and oseltamivir were determined for VN/1203-H5N1-infected cells (Figure 3). VN/1203-H5N1 is highly infectious, causing rapid cell death of infected A549 cells. Therefore, we evaluated drug efficacy in VeroE6 cells, which are more resilient to cell death and are a good model to evaluate drug efficacy. VeroE6 cells were either (A) pre-virus-treated 24 h before infection with VN/1203-H5N1, or (B) treated 1 h after infection with VN/1203-H5N1. Ten-fold dilutions of probenecid or oseltamivir (100,000, 10,000, 1000, 100, 1, 0.1, 0.01, 0.001, or 0 μM) were examined. The IC_50/90_ for pre-virus treatment of VeroE6 cells with probenecid was 0.00003 μM/0.002 µM, and the IC_50/90_ for oseltamivir was 0.004 μM/4.35 μM. The IC_50/90_ for treatment 1 h after infection with probenecid was 0.001 μM/9.36 μM, and for oseltamivir it was 74.2 μM/667.8 μM (Figure 3). The IC_50/90_ showed that probenecid inhibited viral replication better than oseltamivir for either treatment.

**Figure 4:**
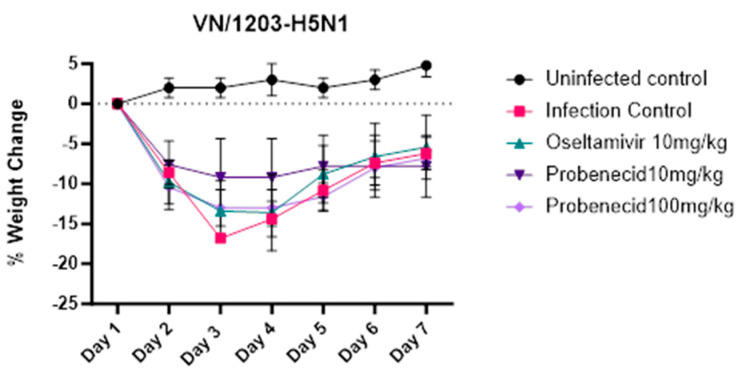
Weight Change. BALB/c mice were anesthetized with avertin and inoculated intranasally with 3LD_50_ of VN/1203-H5N1 (30 µL/mouse). Probenecid (10 or 100 mg/kg) or oseltamivir (10 mg/kg) were administered by oral gavage twice daily for 3 days. Uninfected control mice were gavaged twice daily for 3 days with 100 µL sterile PBS.

**Figure 5:**
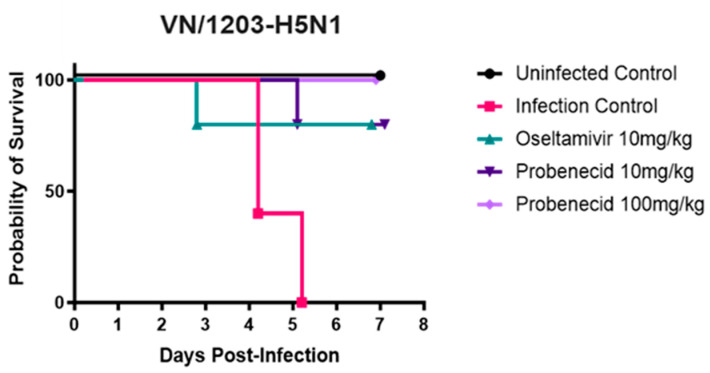
Survival. BALB/c mice were anesthetized with avertin and inoculated intranasally with 3LD_50_ of VN/1203-H5N1 (30 µL/mouse). Probenecid (10 or 100 mg/kg) or oseltamivir (10 mg/kg) were administered by oral gavage twice daily for 3 days. Uninfected control mice were gavaged twice daily for 3 days with 100 µL sterile PBS.

Next, we examined the effect of probenecid or oseltamivir treatment on weight loss (attributed to disease) in VN/1203-H5N1-infected (3LD_50_) BALB/c mice (Figure 4). A 250 μM working stock was prepared in 0.1% DMSO, which is non-cytotoxic. Oseltamivir phosphate, a neuraminidase (NA) inhibitor, is FDA-approved for treating acute, uncomplicated influenza in patients 2 weeks of age and older whose symptoms have not lasted more than 2 days [41]. The FDA-approved dose for oseltamivir is 75 mg taken twice (two times) a day (BID) for 5 days [42]. Probenecid was dosed at 10 mg/kg or 100 mg/kg, while oseltamivir was dosed at 10 mg/kg. All doses were administered via oral gavage twice daily for 3 days (Figure 4). The 10 mg/kg/day oseltamivir dose in mice was chosen because the oral bioavailability is similar to the recommended human oral dose of 75 mg BID twice daily [42]. The oseltamivir dosage was adjusted for the interspecies difference in esterase activity and metabolic rates [43]. The mice were weighed daily for seven days. Mice that lost 20% of their body weight were humanely euthanized. The mice in the infection control (untreated) group had the highest and most rapid percentage of body weight loss, and by day 6 pi, all remaining mice were euthanized. The oseltamivir-treated mice exhibited delayed or slower weight loss but after day 4 pi developed rapid weight loss compared to 10 or 100 mg/kg probenecid-treated mice, which maintained a stable weight at day 3 pi through day 6 pi. The survival of infected and drug-treated mice correlated with body weight loss (Figure 5). The infection control (untreated) mice all succumbed by day 6 pi. Mice treated with oseltamivir or probenecid 10 mg/kg each lost one mouse before the study ended on day 7 pi. Mice treated with probenecid 100 mg/kg all survived.

**Figure 6:**
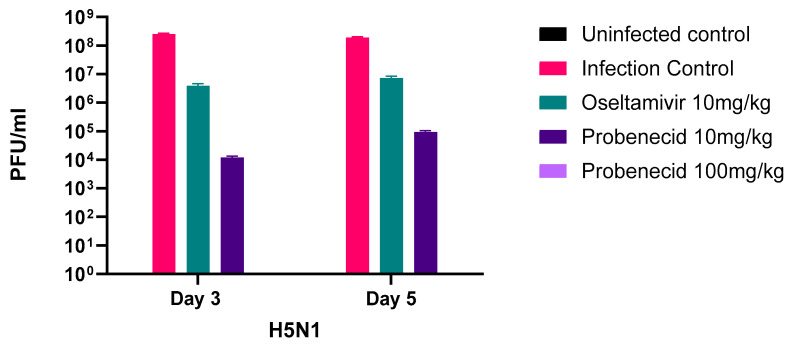
Infectious virus in the lungs. BALB/c mice were anesthetized with avertin and inoculated intranasally with 3LD_50_ of VN/1203-H5N1 (30 µL/mouse). Probenecid (10 or 100 mg/kg) or oseltamivir (10 mg/kg) were administered by oral gavage twice daily for 3 days. Uninfected control mice were gavaged twice daily for 3 days with 100 µL sterile PBS.

The percentage weight loss (Figure 4) closely matched the probability of survival (Figure 5). The amount of infectious virus in the lungs of the infected and treated mice was determined (Figure 6). On days 3 and 5, the lungs were processed, and viral plaque assays were performed. On both days, 3 and 5 pi., the probenecid 100 mg/kg treatment eliminated the lung virus. The 10 mg/kg probenecid treatment reduced the lung virus by 4 logs on day 3 and 3 logs on day 5.

Exacerbated inflammation with H5N1 and H7N9 infection is associated with systemic edema and extensive tissue damage [44]. Analysis of serum from hospitalized patients showed that elevated expression of several pro-inflammatory mediators, including IL-6, is shared following H5N1 and H7N9 infections [45], and analysis of bronchoalveolar lavage from hospitalized H7N9-infected patients showed elevated levels of pro-inflammatory mediators including IL-1β and IL-6 [46]. Proinflammatory cytokine responses induced by H5N1- or H7N9-infected primary human alveolar and bronchial epithelial cells also suggested an exacerbated inflammatory response [47,48]. Interestingly, inhibition of pro-inflammatory cytokines, such as TNF-α and IL-6, does not protect against lethal H5N1 influenza infection in mice [49], suggesting that inflammation alone is likely not the only cause of pathology.

**Figure 7:**
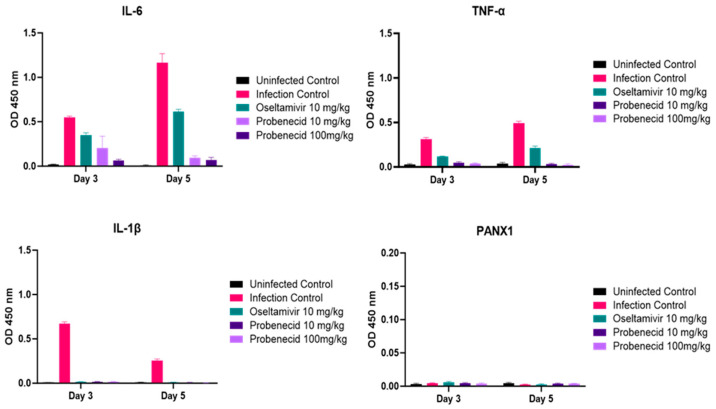
BALB/c mice were anesthetized with avertin and inoculated intranasally with 3LD_50_ of VN/1203-H5N1 (30 µL/mouse). Probenecid (10 or 100 mg/kg) or oseltamivir (10 mg/kg) were administered by oral gavage twice daily for 3 days. Uninfected control mice were gavaged twice daily for 3 days with 100 µL sterile PBS. The cytokines were assayed by ELISA in serum samples of mice obtained on days 3 and 5 pi.

We examined the levels of several proinflammatory (IL-1β, IL-6, and TNF-α) serum cytokines and PANX1 by ELISA. These cytokines have been associated with inflammation from H5N1 and H7N9 infections [48,50,51,52] (Figure 7). Serum from VN/1203-H5N1-infected (3LD_50_) mice were collected at days 3 and 5 pi from 10 mg/kg or 100 mg/kg probenecid-treated, 10 mg/kg oseltamivir-treated, or infection control (untreated) mice. The results showed that on both days 3 and 5, the 10 mg/kg or 100 mg/kg probenecid-treated mice had significantly (*p* < 0.05) reduced IL-6, TNF-α, and IL-1β when compared to the infection control mice. As expected [43], oseltamivir-treated mice had significantly (*p* < 0.05) reduced IL-6, TNF-α, and IL-1β at days 3 and 5 pi. There were significant (*p* < 0.05) differences between probenecid and oseltamivir at day 5 pi for IL-6 and TNF-α expression. Also, as expected, the transmembrane protein PANX1 was undetectable (Figure 7).

## 4. Discussion

Human infections with avian IAVs are uncommon but have occurred. The HPAI subtypes virologically confirmed to have infected people are H5 and H7 viruses. HPAI H5N1 infections have been reported in >890 people, with approximately 50% case fatality [53]. HPAI H7N9 infections have been reported in China since 2016, with the case fatality proportion in hospitalized patients being ~40% [54]. People with HPAI infection are recommended to be treated as soon as possible with antiviral drugs that are FDA-approved for the treatment of seasonal influenza. These antiviral drugs include oseltamivir, zanamivir, peramivir, and baloxavir. Antiviral treatment works best when started as soon as symptoms begin. Several antiviral drugs have been developed to treat influenza viruses, but all have some restricted efficacy against influenza viruses. For example, these antiviral drugs are direct-acting antivirals that target stages of the viral life cycle. M2 inhibitors, like amantadine and rimantadine, which block ion channel activity and prevent the release of the viral genomes into the cell cytoplasm [55], are effective for the IAVs but not for the influenza B virus because they lack the M2 protein [56]. Since NA is required for virus release and regulating receptor binding and virus budding [57], NA inhibitors, such as oseltamivir are generally effective. However, their use usually leads to resistance [58]. Because IAVs accumulate mutations increasing the likelihood of drug evasion [59], new antiviral drugs are needed to keep pace with the continuous IAV variation.

The potential for a host-directed antiviral drug was realized from our studies investigating the host genes required for IAV to replicate in human A549 cells [29,30,31,60,61,62]. In these studies, genome-wide RNA interference (RNAi) screens were performed to determine host genes that were pro- or antiviral and affected virus replication. From these RNAi screens we discovered host genes whose loss of function led to the inhibition of virus replication. Specifically, we showed that the organic anion transporter-3 (OAT3) gene was an important host gene required for virus replication [6]. Thus, we tested probenecid, a pharmacological inhibitor of OAT3, which is FDA-approved and has a decades-long safety profile [63,64].

Probenecid treatment was found to inhibit OAT3 mRNA and reduce protein levels in both infected and uninfected A549 cells [6]. In vitro and in vivo probenecid treatment (nanomolar to micromolar) led to the inhibition of viral replication with probenecid reducing IAV replication with an IC_50_ range of 5 × 10^−5^ to 5 × 10^−4^ μM and in mice treated daily over 3 days with 25 mg/kg probenecid. Probenecid treatment also protected mice from lethal challenge with mouse-adapted A/WSN/33, with a 60% survival rate. Furthermore, probenecid pre-virus treatment was effective against several IAV strains in A549 cells and BALB/c mice. When mice were treated prophylactically with 200 mg/kg of probenecid (24 h before infection), therapeutically with 200 mg/kg probenecid (24 hpi), or with 25 mg/kg probenecid daily for 3 days following infection, they exhibited reduced morbidity and mortality and had low to no lung virus titers. Recent studies have shown that probenecid has pan-antiviral effects against SARS-CoV-2 [5] and RSV [3] replication in vitro and in vivo. Probenecid prophylaxis or treatment inhibited SARS-CoV-2 replication, including variants of concern in Vero E6 and NHBE cells at concentrations as low as 0.00001–100 μM. When hamsters were treated with probenecid 24 h before or 48 h after infection, lung virus titers were greatly reduced, i.e., a 4–5_log_ virus reduction compared to the controls. Probenecid also inhibited RSV replication in human respiratory epithelial cell lines and mice. The greatest reductions in lung virus load occurred in mice that were pretreated with 200 mg/kg probenecid 24 h before infection, while treatment with 2 or 200 mg/kg probenecid 24 h after RSV infection also significantly reduced lung titers on days 3, 5, and 7 pi. Moreover, a Phase 2 randomized, placebo-controlled, single-blind, dose-range finding study on non-hospitalized patients with symptomatic, mild-to-moderate COVID-19 showed that probenecid treatment significantly reduced viral clearance and increased the proportion of patients that completely resolved symptoms compared to the placebo [2]. The pan-antiviral effects of probenecid suggest that its mechanism of action likely involves host cell pathways used by viruses for replication.

The JNK (c-Jun N-terminal kinase) pathway is necessary for the mitogen-activated protein kinase (MAPK) signaling pathway [65,66]. The JNK pathway is activated by extracellular (e.g., inflammatory signals, viruses, etc.) and intracellular stimuli (e.g., oxidative stress and DNA damage). Evidence suggests JNK and extracellular signal-regulated kinases (ERKs) are activated by virus infection [67], and recent Western blot evidence indicates that JNK activates MAPK, which downregulates the expression of hepatocyte nuclear factor 4 alpha (HNF4) [68], and HNF-4 regulates the expression of certain OATs [69]. These emerging data suggest that the mechanism of action of probenecid involves inhibiting JNK phosphorylation and downstream HNF-4 regulation of OAT3, likely inhibiting virus assembly and replication.

Both HPAI H5N1 and H7N9 can cause severe disease in humans with symptoms that include acute respiratory distress syndrome (ARDS) [70]. In vitro and in vivo studies in mice have shown differences between HPAI H5N1 and H7N9 infections in mice, including in viral replication, spread, and the host immune response [71]. Probenecid has been shown to modify PANX1 expression, a ubiquitously expressed, channel-forming protein found in tissues throughout the body, including the lungs, central nervous system, and immune system [72]. There were no detectable differences in serum PANX1 expression detected in this study, which is not surprising, as circulating levels of PANX1 would not be expected to increase, but inhibition of their function would still be expected to produce an anti-inflammatory response. Probenecid treatment may reduce inflammation, as multiple studies have shown that PANX1 signaling exacerbates inflammatory responses due to the secretion of proinflammatory cytokines, such as IL-1β and IL-6 [73,74]. In addition, probenecid has been shown to inhibit the NLRP3 inflammasome response, reduce hyperinflammation, and improve survival associated with severe influenza virus infection [73]. Consistent with the putative mechanism of action of probenecid, JNK has been shown to have an important role in the expression of the proinflammatory cytokines (e.g., TNF-α), and probenecid inhibits JNK phosphorylation, reducing inflammation [75,76,77]. Together, these studies show that probenecid has potent antiviral effects and significantly improves inflammation associated with HPAI H5N1 and H7N9 infection in mice.

## Data Availability

The data supporting the reported results can be found in the Tripp laboratory at the University of Georgia.
